# Cellulose-based bionanocomposites in energy storage applications-A review

**DOI:** 10.1016/j.heliyon.2023.e13028

**Published:** 2023-01-16

**Authors:** Atanu Kumar Das, Md Nazrul Islam, Rupak Kumar Ghosh, Roni Maryana

**Affiliations:** a Department of Forest Biomaterials and Technology, Swedish University of Agricultural Sciences, SE- 90183, Umeå, Sweden; b Forestry and Wood Technology Discipline, Khulna University, Khulna, 9208, Bangladesh; c Forest Chemistry Division, Bangladesh Forest Research Institute, Chittagong, 4211, Bangladesh; d Research Center for Chemistry, National Research and Innovation Agency, South Tangerang, Banten 10340, Indonesia

**Keywords:** Microcellulose, Nanocellulose, Electrode, Battery, Supercapacitor

## Abstract

The growing demand for energy and environmental issues are the main concern for the sustainable development of modern society. Replacing toxic and expensive materials with inexpensive and biodegradable biomaterials is the main challenge for researchers. Nanocomposites are of the utmost consideration for their application in energy storage devices because of their specific electrochemical properties. Cellulose-based bionanocomposites have added a new dimension to this field since these are developed from available renewable biomaterials. Studies on developing electrodes, separators, collectors, and electrolytes for the batteries have been conducted based on these composites rigorously. Electrodes and separators made of these composites for the supercapacitors have also been investigated. Researchers have used a wide range of micro- and nano-structural cellulose along with nanostructured inorganic materials to produce cellulose-based bionanocomposites for energy devices, i.e., supercapacitors and batteries. The presence of cellulosic materials enhances the loading capacity of active materials and uniform porous structure in the electrode matrix. Thus, it has shown improved electrochemical properties. Therefore, these can help to develop biodegradable, lightweight, malleable, and strong energy storage devices. In this review article, the manufacturing process, properties, applications, and possible opportunities of cellulose-based bionanocomposites in energy storage devices have been emphasized. Its challenges and opportunities have also been discussed.

## 1 Introduction

A sustainable supply of energy is the utmost concern to meet the growing energy demand in modern society. A sufficient energy supply is crucial for the sustainable development of society [1,2]. Improved living standards and technological development for electronic devices, sensors, and others urge to generate more energy [3,4]. To meet the energy demand, energy storage and conversion into required forms are important considerations [5–7]. Supercapacitors, electrochemical capacitors, can store electrical energy in the interface of electrodes and electrolytes [8]. Electrochemical energy storage devices, such as supercapacitors [6,9,10], lithium-ion batteries (LIB) [11], zinc-ion batteries (ZIB) [12], and lithium-sulfur batteries (LISB) [13] have a high energy density, capacity retention, and are safe to use. Considering these, researchers have shown their interest in these types of energy storage devices [9].

However, a conventional supercapacitor is composed of expensive and toxic components [[14], [15]]. Furthermore, technical issues of LIB and LISB are a hindrance to meeting the growing energy demand for these batteries [16]. Therefore, an environmentally friendly supercapacitor [17] and batteries [16] are necessary to meet the sustainable energy supply. Renewable bio-based materials can replace hazardous materials [18]. These are promising to develop functional composites, which possess unique microstructure and electrochemical properties [19]. In addition, developing nanostructured energy devices is a growing interest for researchers concerning energy and environmental issues [11]. Nanostructured composite materials enhance the electrochemical properties of energy devices [13,20,21].

Cellulose, the most available renewable bio-based material, contains >50% carbon content in the plant kingdom [19]. Cellulose-based bionanocomposites have been applied in supercapacitors [1,22,23] and batteries [16]. Cellulose and cellulose derivatives, i.e., carboxymethyl cellulose (CMC), cellulose acetate (CA), and nanocellulose (NC), mainly work as binder and dispersing agents in various applications [18]. These can also be used as aerogel and carbon aerogel in making an electrode for energy storage devices [24]. Layer-by-layer (LBL) is the advanced technique for improving the properties [25], and this technique has been used developing a separator for LIB [26]. Researchers have also applied papermaking technique to make a separator from the cellulose-based bionanocomposite for LIB [27]. Besides these, an electrolyte for LIB [28] and collector for lithium silicon battery (LISiB) [29] have been produced as energy storage devices. Electrode [30] and electrolyte [31] for supercapacitor from cellulose-based bionanocomposites have also been investigated. However, there is no single review work that emphasizes cellulose-based bionanocomposites in energy storage applications. In previous review studies, researchers have targeted nanocellulose-based nanocomposites in energy storage applications [32,33]. There is lacking information on cellulose-based bionanocomposites and their applications in energy storage devices.

Therefore, this review work was conducted to provide the state of the art of utilization of cellulose-based bionancomposites in developing energy storage devices. Solid-state bionanocomposites were focused in this review work. Types of cellulose and its derivatives, manufacturing processes, properties, and applications in energy storage devices development were discussed. Challenges and opportunities for the implications of cellulose-based bionancomposites in energy storage devices were also pointed out in this review article. The basic information regarding the bionanocomposites and their applications in energy storage devices was also mentioned in the beginning.

## 2 Bionanocomposite and cellulose-based bionnanocomposite

Biocomposites are composed of biopolymers, i.e., lignin, hemicellulose, and cellulose [34], and bio-based materials [[34], [35], [36], [37]]. These composite materials also possess inorganic materials in their matrix [38]. Again, nanocomposites contain at least one material with one dimension on a nanometer scale [38,39]. Therefore, bionanocomposites are made of at least one biopolymer or bio-based material having any material with nanoscale dimension in the matrix. Cellulose-based bionanocomposites are composed of cellulose and its derivative along with organic or inorganic material in which one material is nanoscale in dimension. Cellulose in micro and nanoscale is used in developing cellulose-based bionanocomposites. The main source of cellulose is plants, and it is also extracted from bacteria. Cellulose derived from bacteria is known as bacterial cellulose (BC). Cellulose in the nanoscale derived from plant is called nanocellulose (NC), and it has been classified into different types [40]. On the other hand, BC on the nanoscale is referred to as bacterial nanocellulose (BNC). These nanocomposites are advanced hybrid materials in composite science and technology.

## 3 Cellulose-based bionanocomposites for energy storage applications

**Fig. 1:**
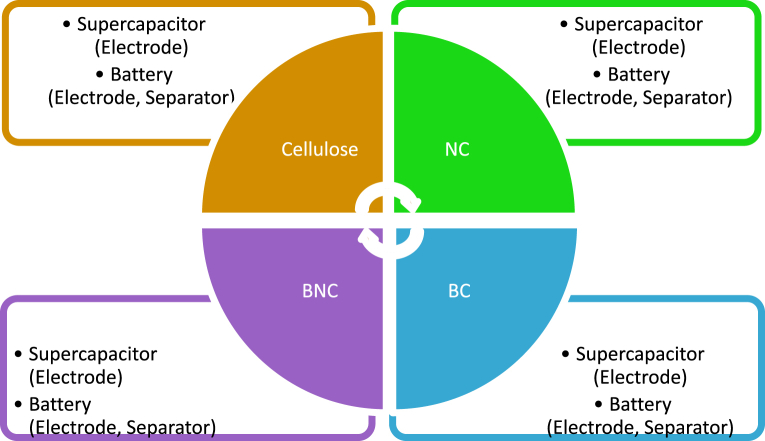
An overview of the applications of cellulose-based bionanocomposites in energy storage devices.

Cellulose and its derivatives sourced from plants and bacteria in micro and nanostructure have been used to develop cellulose-based bionanocomposites for the implication in energy storage devices. These composite materials have been used to prepare the electrodes, i.e., cathode and anode, separator, and electrolyte for a battery and a supercapacitor (Fig. 1). These materials possess special characteristics to improve their electrochemical properties. For example, carboxymethyl cellulose (CMC) can form a dendritic network structure via charge-charge interactions through cross-linking the conductive polymer chains, which help to enhance the electrochemical properties [41]. In addition, nanoscale structural features of nanocomposites enhance the specific capacity [13] and capacity retention [20] of the battery. Therefore, researchers have shown great interest in developing green energy storage devices using cellulose-based bionanocomposites.

## 4 Cellulose-based bionanocomposites for batteries

Plant and bacteria-based cellulose in micro-and nanostructure have been applied for developing bionanocomposites for batteries. Cellulose-based bionanocomposite can be used as an electrode, electrolyte, and separator for the batteries [33]. It can also be used as a collector for the batteries. An overview of the implications of cellulose-based bionanocomposites for the batteries has been presented in Table 1.

**Table 1:** Cellulose based bionanocomposites and their properties for batteries.

Cellulose	Fabrication	Bionanocomposite	Battery component	Battery type	Electrolyte	Electrochemical properties			Ref
						Capacitance	Capacitance retention	Energy density	
CF	Hydrothermal and vacuum filtration	CF/CNT/rGO/NVO	Electrode (cathode)	ZIB	2.0 M ZnSO_4_	1.87 mAh cm^-2^	92% after 4000 cycles	295.4 Wh kg-1 at 162.3 W kg^-1^	[12]
CF	Hydrothermal and calcination	CF/NiO	Electrode (Anode)	LIB	1.0 M LiPF_6_ dissolved in a mixture of EC and DEC	476 mA h g^−1^	727 mA h g^−1^ after 150 cycles at 100 mA g^−1^		[19]
CF	sol gel process and carbonization	CF/SnO2	Electrode (Anode)	LIB	1.0 M LiPF_6_ dissolved in a mixture of DMC and EC	555 mA h g-1 at 50 mA g-1			[45]
CF	Papermaking and vacuum filtration	CF/SA/Si	Separator	LIB	1.0 M LiPF_6_ dissolved in a mixture of EC and DMC		75% after 200 cycles		[64]
EC	Vacuum filtration	CF/MWCNT	Collector	LISiB	1 M LiPF_6_ dissolved in a mixture of EC and DEC		900 mAh g^-1^ after 200 cycles at 200 mA g^-1^		[29]
Na-CMC	Freeze drying and carbonization	Na-CMC/SnO2/GO	Electrode (Anode)	LIB	1.0 M LiPF_6_ dissolved in a mixture of EC, DEC, and VC		1458.8 mA h g^-1^ remaining after 700 cycles at 1.0 A g^-1^		[47]
CA-CMC	Coating	CA-CMC/MWCNT/S/KB	Electrode (cathode)	LISB	1.0 M LiTFSI dissolved in a mixture of LiNO_3_, DOL, and DME		960 mAh g^-1^ after 200 cycles at 0.1C		[50]
CMC	Hydrothermal and coating	CMC/SnO2/MWCNT	Electrode (Anode)	LIB	1.0 M LiPF_6_ dissolved in a mixture of EC and DEC		473 mA h g^−1^ after 100 cycles		[48]
CMC	Hydrothermal, calcination, and coating	CMC/Mn/Co/SBR/Cu	Electrode (Anode)	LIB	1.0 M LiPF_6_ dissolved in a mixture of EC, DEC, and FEC		1364.47 mAh g^-1^ at 0.1 A g^-1^ after 100 cycles		[49]
EC	sol gel and carbonization	EC/Si	Electrode (Anode)	LIB	1.0 M LiPF_6_ dissolved in a mixture of EC, DEC, and FEC		92% (1830 mAh g^-1^ at 200 mA g^-1^ after 60 cycles)		[57]
CMC	self-assembly process and thermal condensation reaction	CMC/rGO/Si/PAA	Electrode (Anode)	LIB	1.0 M LiPF_6_ dissolved in a mixture of DMC and EC	2153.49 mA h g^-1^	63% after 800 cycles at and 420 mA g^-1^		[51]
MCC	Pyrolysis and melt infiltration	MCC/S	Electrode (cathode)	LISB			600 mA h g-after 200 cycle		[61]
CNF	Roll-to-roll	CNF/MoS2/CNT	Electrode (Anode)	SIB			147 mAh g-1 after the first cycle		[60]
CNF	Sol-gel process followed by carbonization and magnesiothermic reduction technique	CNF/Si/TiO	Electrode (Anode)	LIB	1.0 M LiPF_6_ dissolved in a mixture of EC, EMC, and DEC		792.6 mAh g^-1^ at 100 mA g^-1^ after 160 cycles		[11]
CNF	in situ polymerization and LBL	CNF/CNF/PPy	Separator	LIB		161 mA h g ^−1^ at 0.2C			[26]
CNF	Vacuum filtration	CNF/ZIF8	Separator	LIB	1.0 M LiPF_6_ dissolved in the mixture of EC and DMC		88.3%		[65]
BC	Hydrothermal and carbonization	BC/MoS_2_	Electrode (Anode)	LIB	1.0 M LiPF6 dissolved in a mixture of EC and DMC	1137 mA h g^-1^ at 1C	967 mA h g^-1^ after 50 cycles at 1C		[62]
BNC	Hot bath and carbonization	BNC/SnS_2_	Electrode (Anode)	LIB	11.0 M LiPF6 dissolved in a mixture of EC and DMC		872 mA h g^-1^ at 100 mA g^-1^ after 100 cycles		[63]
BC	Paper-making	BC/ANFs	Separator	LIB			93% after 100 cycle		[27]
MCCF and NC	Casting	MCCF/NC	Electrolyte	LIB			92% after 50 cycles		[28]

### 4.1 Electrode of cellulose-based bionanocomposites for batteries

#### 4.1.1 Plant oriented cellulose-based bionanocomposites as electrode for batteries

##### 4.1.1.1 Microstructural cellulose

**Fig. 2:**
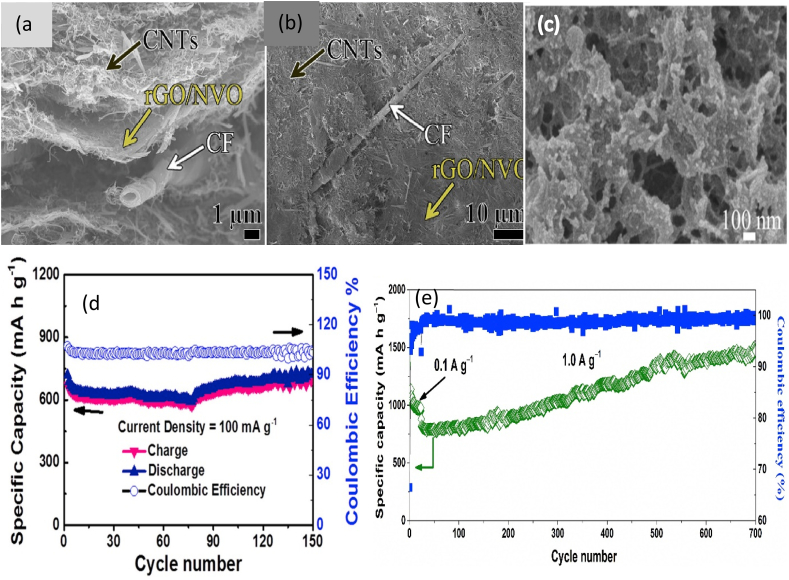
(a) Cross-sectional [12] and (b) Surface SEM images of CF/CNT/rGO/NVO electrode [12]; (c) SEM image of CF/NiO electrode [19]; (d) Capacity versus cycle numbers at the current density of 100 mA g−1 and the corresponding Coulombic efficiency of CF/NiO electrode [19]; and (e) Long-term cycle performance and corresponding coulombic efficiency of the Na-CMC/SnO_2_/GO electrode [47].

Cellulose fiber (CF) has been used to fabricate the nanocomposite as an electrode for ZIB (Fig. 2a and b) [12]. Reduced graphene oxide (rGO) and δ-Na_x_V_2_O_5_·nH_2_O (NVO) were hydrothermalized to produce rGO/NVO nanocomposite. It was then mixed with carbon nanotube (CNT), CF, and water to obtain the slurry followed by vacuum filtering and washing to obtain the nanocomposite of CF/CNT/rGO/NVO as an electrode. It showed the specific capacity, charge density, and capacity retention of 1.87 mAh cm^-2^, 295.4 Wh kg^-1^ at 162.3 W kg^-1^, and 92% after 4000 cycles, respectively in 2.0 M ZnSO_4_ electrolyte. CF reduces charge transfer hindrance and increases the Zn^2+^ ion diffusion coefficient and pseudocapacitive intercalation because CF provides excellent electrochemical properties [12]. Similarly, the use of CF for making CF/NiO nanocomposite (Fig. 2c) based electrode (anode) for LIB showed high charge and electron transfer efficiency [19]. It was fabricated by hydrothermal and air calcination methods. The mixture of CF, urea dissolved in ethylene glycol, and NiSO_4_•6H_2_O was hydrothermalized followed by filtering, cleaning, drying, and calcination to produce CF/NiO nanocomposite. It was then used as an anode in LIB and 1.0 M lithium hexafluorophosphate (LiPF6) dissolved in ethylene carbonate (EC) and diethyl carbonate (DEC) was worked as an electrolyte. The current density and specific capacity were 2 A g^−1^ and 476 mA h g^−1^, respectively. Capacity retention was 727 mA h g^−1^ after 150 cycles at 100 mA g^−1^ (Fig. 2d). The unique nanostructure of the CF/NiO provides an interconnected network with nanopores and a high specific surface area leading to high Li^+^ storage performance and electrochemical properties [19]. In another study, in situ regenerating technique was applied to obtain CF/CNT/Si and CF/CNT/Fe_3_O_4_ bionanocomposite as an electrode (anode) [42]. Again, researchers embedded multiwalled carbon nanotube (MWNT) into cellulose paper to produce electrodes as cathodes for LIB [43]. Zhou et al. [44] produced CF/GE/MWCNT/Cu-based electrodes (anode) for LIB by vacuum filtration, carbonization, and coating. The electrolyte was 1.0 M LiPF6 dissolved in a mixture of DMC and EC. The cyclic stability and coulomb efficiency were 438 mA h g^−1^ and 50.2%, respectively. Oh et al. [45] used the carbonization technique along with the sol-gel process to prepare CF/SnO_2_ bionanocomposite as an electrode (anode) for LIB. The authors used a similar electrolyte to Zhou et al. [44]. The battery showed a specific capacity of 555 mA h g^-1^ at 50 mA g^-1^. The carbonization enhances graphitization and carbon cluster formation, which add value to obtaining excellent electrochemical properties [44]. The incorporation of CF increases the loading of SnO_2_, produces the 3D network, and reduces volume expansion leading to excellent electrochemical properties. It also provides a uniform porous structure for transferring electrons [45].

As nanostructure has the potential to improve the electrochemical properties, researchers have observed the application of tin nanoparticles along with carbon material as an anode can increase the energy density of LIB [46]. For this, researchers used sodium carboxymethyl cellulose (Na-CMC) and SnO_2_ nanoparticles to prepare an electrode (anode) for LIB [47]. The authors freeze-dried the mixture of SnO_2_ nanoparticles coated glucose (hydrothermal process), GO, ethanol, and Na-CMC and poly (vinyl alcohol) (PVA) followed by carbonization to obtain the Na-CMC/SnO_2_/GO bionanocomposite. Here, Na-CMC worked as a binding material. It was then employed as an anode in LIB and the electrolyte was 1.0 M LiPF_6_ dissolved in a mixture of EC, DEC, and vinylene carbonate (VC). The electrode showed the rate capability and cycling stability (Fig. 2e) of 611.1 mA h g^-1^ at 4.0 A g^-1^ and 1458.8 mA h g^-1^ remaining after 700 cycles at 1.0 A g^-1^, respectively [47]. Noerochim et al. [48] also observed the excellent electrochemical properties of CMC-based electrodes as an anode in LIB. In this type, researchers coated Cu foil using CMC/SnO_2_/MWCNT bionanocomposite followed by vacuum drying and pressurizing to prepare the electrode. In the beginning, the authors prepared SnO_2_/MWCNT nanocomposite by hydrothermalization of the mixture of SnCl_4_·5H_2_O, water, and acid-treated MWCNT. It was then mixed with acetylene black (AB), CMC, and a solvent dissolved in water before the coating of Cu foil. The used electrolyte was 1.0 M LiPF_6_ dissolved in a mixture of EC and DEC for this study. The capacity retention was 473 mA h g^−1^ after 100 cycles. In another study, Muruganantham et al. [49] coated the Cu foil using CMC-based nanocomposite to prepare the electrode (anode) for LIB. For this, CoV_2_O_4_ nanoparticles were modified with Mn using hydrothermal and calcination. Then, these were mixed with carbon black, CMC, and styrene butadiene rubber (SBR) to obtain the bionanocomposite slurry, which was used to coat the Cu foil to prepare the electrode of CMC/Mn/Co/SBR/Cu. The specific capacity was 1364.47 mA h g^-1^ at a current density of 0.1 A g^-1^ after 100 cycles in the electrolyte of 1 M LiPF6 dissolved in a mixture of EC, DEC, and fluoroethylene carbonate (FEC). The modification of Co with Mn lowers the bandgap. In addition, the presence of nanoparticles, high specific surface area, and porous structure increases the electrical conductivity and performance of electrochemical properties [49]. Other researchers coated carbon paper or carbon-coated aluminium foil by acid-CMC (CA-CMC)-based nanocomposite to produce an electrode (cathode) for LISB [50]. The authors made a slurry of CA-CMC, MWCNT, S, and Ketjenblack (KB) by grinding, heating, and mixing with water for the coating. The electrolyte was 1 M bis(-trifluoromethanesulfonyl)imide (LiTFSI) with 2 wt% LiNO_3_ dissolved in 1,3-dioxolane (DOL) and dimethoxymethane (DME). LISB showed a capacity of 960 mA h g^-1^ after 200 cycles at 0.1 C. Wang et al. [51] used CMC, rGO, Si nanoparticle, and polyacrylic acid (PAA) to produce bionanocomposite of CMC/rGO/Si/PAA as an electrode (anode) for LIB using a self-assembly process and thermal condensation reaction. The electrolyte was a 1 M solution of LiPF6 dissolved in a mixture of dimethyl carbonate (DMC) and EC. The capacity and capacitance retention of LIB was 2153.49 mA h g^-1^ and 63% after 800 cycles at 420 mA g^-1^, respectively. Na-CMC and CMC enhance the attachment of active materials in the matrix of the electrode [47,48] and reduce the pulverization of the electrode [47,52]. The presence of hydroxyl and carboxylate groups makes an H-bonding with biopolymer to having a rigid structure [53,54]. Furthermore, the structure of the nanocomposite provides an interconnected graphene network with a porous structure and this allows easier electron and lithium ion transport [47]. The uniform distribution of active material in the matrix enhances the structural stability and electrochemical properties of the electrode. In addition, the amount of CMC content influences the porosity and interconnection of the composite materials. Thus, it influences the electrochemical properties [50,55].

Ethyl cellulose (EtC) was also used to produce an electrode (cathode) for LISB [13]. The authors prepared graphene (GE) and EtC-based nanocomposite of EC/GE. Then, sulfur-loaded metal−organic framework (MOF/S), EtC/GE, and poly(vinylidene difluoride) (PVDF) were mixed to obtain the nanocomposite of EC/GE/MOF/S. GE has high conductive properties [56], and EtC increases the volumetric capacity of the battery by providing conformal interfaces between GE and cathode particles. Nulu et al. [57] produced EtC/Si-based anode by sol-gel and carbonization process for LIB. The capacity retention was 92% (1830 mAh g^-1^ at 200 mA g^-1^ after 60 cycles) when the electrolyte was 1 M LiPF_6_ dissolved in the mixture of EtC, DEC, and FEC. The EtC worked as conductive and helped to remain the inherent properties of the anode through coupling with Si nanoparticles.

##### 4.1.1.2 Nanostructural cellulose

**Fig. 3:**
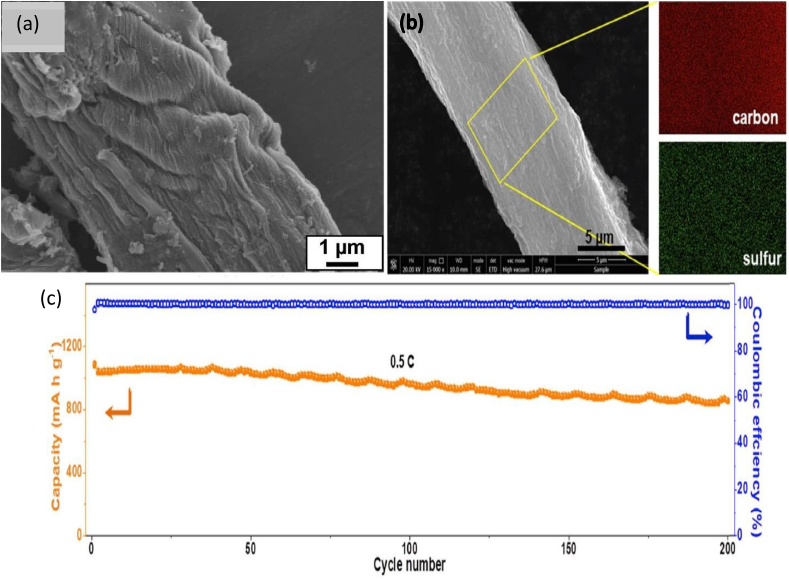
(a) SEM image of CNF/Si/TiO nanocomposite [11]; (b) SEM and EDX mapping image of MCC/S nanocomposite [61]; and (c) Capacity of MCC/S based LISB [61].

Researchers have studied the performance of NC-based nanocomposites as an electrode for a battery. CNF and rGO were used to produce CNF/rGO bionanocomposite as an anode of LIB. The authors used the wet-spinning method followed by carbonization. It showed a high conductivity of 649 ± 60 S cm^-1^ and a stable discharge capacity of 312 mA h g^-1^ [58]. In another study, silica (Si) gel and titania (Ti) gel were deposited on the CNF of a filter paper consecutively using a sol-gel process followed by carbonization and magnesiothermic reduction technique to produce CNF/Si/TiO nanocomposite (Fig. 3a) [11]. It was then employed as an electrode (anode) of a LIB where 1.0 M LiPF_6_ dissolved in a mixture of EC, ethyl methyl carbonate (EMC), and DEC was used as an electrolyte. The performance of this LIB was capacity retention of 792.6 mA h g^-1^ at a current density of 100 mA g^-1^ after 160 cycles. Cao et al. [59] used 3D printing technology to prepare the anode from carbonized CNF and lithium (Li). CNF/Li electrode had a capacitance of 2346 mA h g^-1^ and capacitance retention of 85% after 3000 cycles. The same authors produced cathodes from carbonized CNF and LiFePO_4_ (LFP) using the same technology. CNF/LFP cathode showed similar capacitance retention of the anode with the lower value of capacitance of 167 mA h g^-1^. In another study, TEMPO-oxide CNF, molybdenum disulfide (MoS_2_), and carbon nanotube (CNT) were blended with an appropriate ratio to make a CNF/MoS_2_/CNT electrode as an anode for sodium-ion battery (SIB) using a roll-to-roll process. It had a discharge capacity of 147 mAh g^-1^ for the first cycle [60]. The ion accessibility increases due to having a porous structure of CNF leading to high electrochemical properties of the composite materials [59]. Sun et al. [61] produced electrodes (cathode) from microcrystalline cellulose (MCC) and sulfur (S) for lithium-sulfur batteries (LISB) (Fig. 3b). The authors pyrolysed the mixture of MCC and nano-sized SiO_2_ followed by the removal of SiO_2_ and loading of S to obtain the nanocomposite of MCC/S. The nanocomposite electrode exhibited a reversible capacity of 600 mA h g^-1^ after 200 cycles (Fig. 3c). The large mesopore size with high surface area works as a host of S, which empowers the electrochemical properties of the bionanocomposite materials.

#### 4.1.2 Bacterial cellulose-based bionanocomposites as electrode for batteries

##### 4.1.2.1 Microstructural cellulose

Li et al. [62] synthesized BC and MoS_2_ nanosheet-based nanocomposite for use as an electrode (anode) in LIB by hydrothermal and carbonization process. A mixture of BC hydrogel, ammonium molybdate tetrahydrate, and thiourea was autoclaved followed by washing, freeze-drying, and carbonization to obtain the product of BC/MoS_2_ bionanocomposite. It was then used as an electrode for a LIB using an electrolyte of 1 M LiPF_6_ dissolved in EC and DMC. The battery showed a high initial capacity of 1137 mA h g^-1^ at 1C, good cycling stability of 967 mA h g^-1^ after 50 cycles at 1C, and excellent rate performance.

##### 4.1.2.2 Nanostructural cellulose

**Fig. 4:**
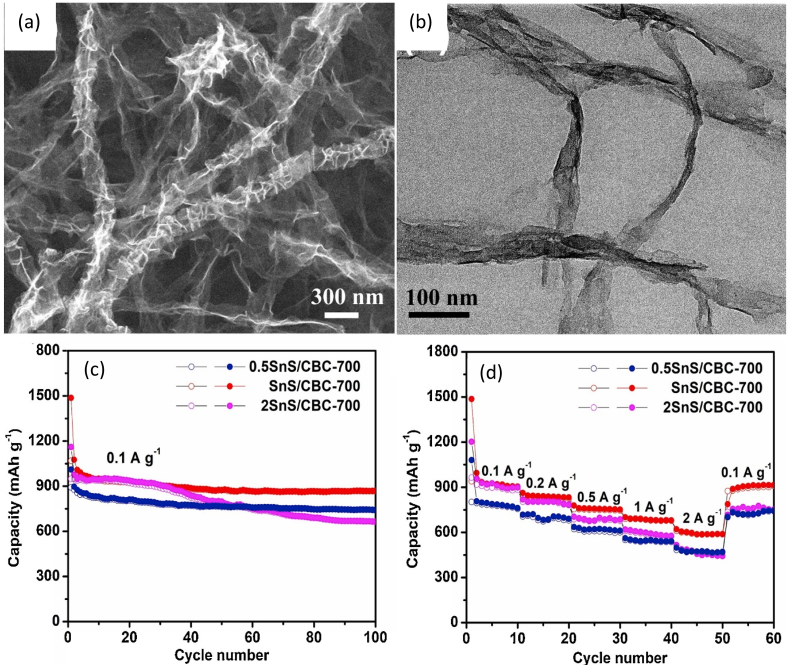
(a) FE-SEM image of BNC/SnS2 nanocomposite; (b) TEM image of BNC/SnS2 nanocomposite; (c) cycling performance of BNC/SnS2 anode at 100 mA g−1; and (d) rate capacities of the BNC/SnS2 anode in the range of 0.1–2 A g−1 [63]. The number 700 refers to carbonization temperature in degree Celsius, and CBC and SnS refer to BNC and SnS2, respectively.

The hot bath method followed by the carbonization process was applied to produce a nanocomposite from tin disulphide (SnS_2_) and BNC as an electrode (anode) for a LIB (Fig. 4a and b) [63]. In this process, the authors soaked rectangular BNC aerogel into the mixture of SnCl_4_, thioacetamide (TAA), and ethanol followed by washing and freeze-drying to obtain BNC/SnS_2_ film. It was then carbonized to produce a free-standing film of BNC/SnS_2_. LIB using BNC/SnS_2_ film as an anode showed the retention capacity of 872 mA h g^-1^ at 100 mA g^-1^ after 100 cycles (Fig. 4c and d) where electrolyte was 1 M LiPF_6_ dissolved in EC and DMC. The uniform distribution of SnS_2_ and MoS_2_ nanosheets in the BNC matrix and the unique porous structure of the composite allow transporting of electrons/Li^+^ leading to high electrochemical properties [62,63]. The high aspect ratio and presence of functional groups of BNC cause anchoring of the metal nanoparticle and the carbonization technique enhance the uniform distribution of them in the bionanocomposite matrix [63]. These are the factors for empowering the electrochemical properties of BNC-based nanocomposite electrodes.

### 4.2 Separator of cellulose-based bionanocomposites for batteries

#### 4.2.1 Separator of plant oriented cellulose-based bionanocomposites for batteries

##### 4.2.1.1 Microstructural cellulose

CF has been used to prepare the separator for LIB [64]. The authors used CF, flame retardant, sodium alginate (SA), and Si nanoparticles to obtain the CF/SA/Si bionanocomposite by papermaking and volume filtration process. LIB showed capacity retention of 75% after 200 cycles when the electrolyte was 1.0 M LiPF6 dissolved in EC and DMC. The improvement of electrolyte uptaking, interface stability, and ionic conductivity provides high electrochemical properties.

##### 4.2.1.2 Nanostructural cellulose

NC-based nanocomposites as separators have been studied for a battery. CNF and Zeolitic imidazolate framework-8 (ZIF8) were used to make CNF/ZIF8 bionanocomposites by vacuum filtration as a separator of LIB [65]. The electrolyte was 1.0 M LiPF6 dissolved in the mixture of EC and DMC. It showed better properties of thermal stability, mechanical property, thermal expansion, and surface wettability compared to a polymer-based separator. LIB composed of CNF/ZIF8 nanocomposites showed better discharge retention stability and comparable cycling stability (88.3% vs 80.2%) compared to commercial polymer membrane-based LIB. LBL technique was also applied to produce polypyrrole (PPy) and CNF-based CNF/PPy bionanocomposite membrane as a separator using the vacuum filtration technique [26]. In this case, PPy was polymerized on CNF by in situ polymerization technique. Then, CNF and CNF/PPy layers were as bottom and top layers, respectively to obtain CNF/CNF/PPy nanocomposite followed by punching. The capacity of LIB was 161 mA h g^−1^ at 0.2C. CNF/PPy layer enhances the mechanical property of the CNF layer and the capacity of LIB.

#### 4.2.2 Separator of bacterial cellulose-based bionanocomposites for batteries

Microstructural BC has been reported for application as a separator. BC blended with aramid nanofibers (ANFs) was used to make a BC/ANFs bionanocomposite membrane by the paper-making process. This membrane exhibited a promising source of separator for LIB in terms of electrochemical properties. The capacity retention of LIB was 93% after 100 cycles [27].

### 4.3 Electrolyte of plant oriented cellulose-based bionanocomposites for batteries

Plant-based cellulose has been reported for application as an electrolyte. The blend of micro carboxylated cellulose fibril (MCCF) and NC obtained from carboxylated cellulose was cast to make a film. It was then converted into a circular film by punching. These were then immersed into 1.0 M LiPF6 dissolved in EC, DMC, and EMC to obtain MCCF/NC electrolyte. The capacity retention of LIB was 92% after 50 cycles, and the ionic conductivity was 1.84 mS cm^-1^. The porous structure of MCCF enhances the transportation of Li^+^ and NC provides structural stability [28].

### 4.4 Collector of plant oriented cellulose-based bionanocomposites for batteries

Plant-based cellulose has been used for the collector of the battery. Researchers used CF for preparing collector [29]. The authors prepared CF/MWCNT based collector by vacuum filtration for Lithium silicon battery (LISiB). However, the authors used an electrode (anode) of CF/MWCNT/Si by coating a slurry of MWCNT and Si on CF/MWCNT. The battery using 1.0 M LiPF6 dissolved in a mixture of EC and DEC as electrolyte showed the capacity retention of 900 mA h g^-1^ after 200 cycles at 200 mA g^-1^ and high coulomb efficiency. The presence of pores and interconnected channels works as the host of Si nanoparticles leading to high conductive performance.

## 5 Cellulose-based bionanocomposites for supercapacitor

Like batteries, cellulose and its derivatives sourced from plants and bacteria have also been used to prepare the bionanocomposites for the supercapacitors. Micro- and nano-structural cellulose have been applied to develop the components of supercapacitors. An overview of cellulose-based bionanocomposites for applications in supercapacitors has been presented in Table 2.

**Table 2:** Cellulose-based bionanocomposites and their properties for supercapacitors.

Cellulosic material	Process	Bionanocomposite	Supercapacitor component	Electrolyte	Electrochemical properties	Capacitance retention	Energy density	Ref
					Capacitance			
CF	In situ polymerization. and electrodeposition	CF/PANI/Ag	Electrode	6.0 M KOH	217 F g^-1^ at a current density of 0.1 A g^-1^	83% after 1000 cycles		[66]
MC	Chemical deposition	MC/CoFe2O4	Electrode (Cathode)	6.0 M KOH	433.3 F g-1 at the current density of 1 A g-1	89% after 2000 cycles	73 W h Kg-1	[70]
CF	Deposition and in situ polymerization	CF/MnO2/PANI	Electrode (Cathode)	PVA dissolved in 1.0 M H_2_SO_4_	103 F g^−1^ in 0.75 A g^−1^	78% after 3000 cycles		[67]
	vacuum filtration and freeze-drying	CF/rGO	Electrode (anode)					
CF	Infiltration and freeze-drying	CF/rGO	Electrode (Cathode and anode)	6 M KOH	255 F g^-1^ at 10 mV s^-1^			[68]
				Room temperature ionic liquid (RTIL)	78 F g^-1^ at 10 mV s^-1^			
CMC	Sol-gel and drying process	CMC/CS/CNT	Electrolyte		94 F g^-1^ at a current density of 100 μA cm^-2^			[31]
NC	Carbonization and in situ microwave depsotion	NC/MnO_2_	Electrode (cathode)	1.0 M Na_2_SO_4_		89% after 10000 cycles	28.2 Wh kg-1	[89]
CNF	Carbonization and coating	CNF/Ni	Electrode	6 M KOH			4.5 Wh kg-1	[92]
NC	Sonication	NC/CNT/PS	Electrode	1.0 M H_2_SO_4_	65 F g^-1^	60% after 2000 cycles		[85]
CNF	Sol-gel	CNF/CNT/PVAB	Electrode (Cathode and anode)	CNF/PVAB hydrogel	117.1 F g^-1^	96.4% after 1000 cycles		[72]
CNF	Vacuum filtration and chemical reduction	CNF/rGO/PPy	Electrode	1.0 M H_2_SO_4_	625.6 F g^-1^ at 0.22 A g^-1^	75.4% after 5000 cycles	21.7 Wh kg^-1^ at 0.11 kW kg-1	[73]
BC	Freeze-drying	BC/AgNps/PANI	Electrode (Cathode and anode)	1.0 M H_2_SO_4_			34 Wh kg^−1^ at 459 Wh kg^−1^	[93]
BC	Freeze-drying	BC/GE/PANI	Electrode (Cathode and anode)	1.0 M H_2_SO_4_			14.2 Wh kg^−1^ at 200 W kg^−1^	[95]
BNC	Vacuum filtration	BNC/PPy/rGO	Electrode (Cathode and anode)	1.0 M NaNO_3_	1.67 F cm^-2^		0.23 mWh cm^-2^ at 23.5 mW cm^-2^	[99]
BNC	Vacuum filtration	BNC/PPy	Electrode (Cathode and anode)	PVDF-EMIMBF_4_	153 F g^-1^	93% after 100 cycles	21.22 Wh kg^-1^ at 0.2 A g^-1^	[100]

### 5.1 Electrode of cellulose-based bionanocomposites for supercapacitor

#### 5.1.1 Plant oriented cellulose-based bionanocomposites as electrode for supercapacitor

##### 5.1.1.1 Microstructural cellulose

**Fig. 5:**
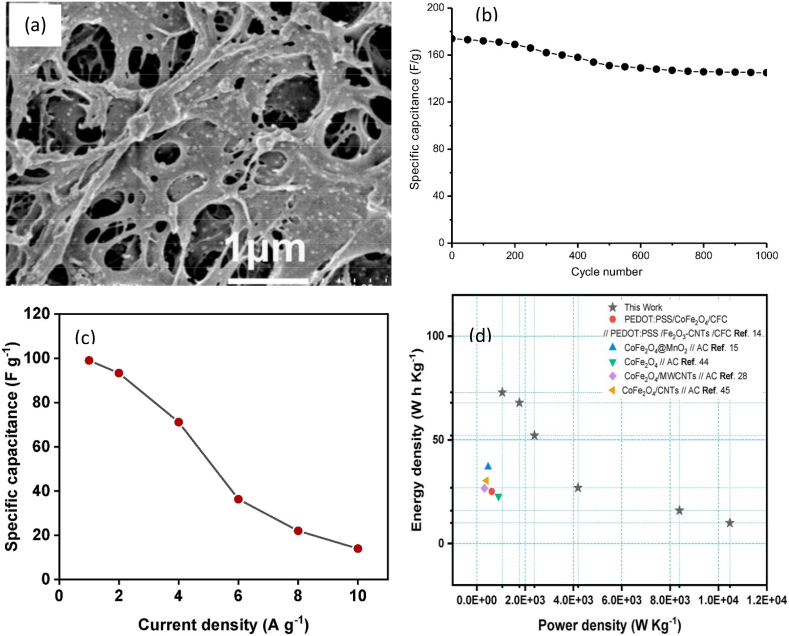
(a) CF/PANI/Ag Electrode [66]; (b) Capacity retention of CF/PANI/Ag electrode at a current density of 0.5 A g^−1^ [66]; (c) Specific capacity [70]; and (d) Power density of MC/CoFe_2_O_4_ electrode-based supercapacitor [70].

Researchers prepared CF/polyaniline (PANI)/Ag nanocomposite aerogel as an electrode for supercapacitors (Fig. 5a) [66]. The authors prepared CF/PANI composite by regeneration of CF followed by in situ polymerization. Then, Ag nanoparticles were electrodeposited on the CF/PANI composite to produce CF/PANI/Ag bionanocomposite as an electrode. The used aqueous electrolyte for electrochemical properties was 6.0 M potassium hydroxide (KOH). The specific capacitance and capacitance retention (Fig. 5b) were 217 F g^-1^ at a current density of 0.1 A g^-1^ and 83% after 1000 cycles, respectively. Hekmat et al. [67] developed a cathode electrode of CF/MnO_2_/PANI following the same technique mentioned above. The specific capacity and capacity retention were 103 F g^−1^ at 0.75 A g^−1^ and 78% after 3000 cycles, respectively when the electrolyte was PVA dissolved in 1.0 M H_2_SO_4_. Again, CF and functionalized nano GO were used to produce CF/GO bionanocomposite for a supercapacitor [8]. The authors produced CF/GO film followed by coating it with gold and attaching it with PET film to obtain the electrode. In another study, CF/rGO electrodes (cathode and anode) were prepared by infiltration of cellulose into the rGO nanosheet followed by freeze-drying [68]. Meanwhile, an N-doped CF/MWCNT/GO electrode was prepared by in situ hydrothermal processes [69]. In the presence of 6.0 M KOH electrolyte, CF/rGO and CF/MWCNT/GO electrodes showed a specific capacity of 255 F g^-1^ at 10 mV s^-1^ [68] and 264 F g^-1^ at a current density of 6 A g^-1^ [69], respectively. CF provides porous scaffolds to capture particles in the matrix [66] and easier access to electrolytes [67] leading to excellent electrochemical properties.

Haghshenas et al. [70] prepared methylcellulose (MC) and CoFe_2_O_4_ nanoparticle-based nanocomposite of MC/CoFe_2_O_4_ as an electrode (cathode) by a chemical deposition method. The used aqueous electrolyte for electrochemical properties was 6.0 M KOH. The specific capacitance (Fig. 5c), energy density (Fig. 5d), power density, and capacity retention were 433.3 F g^-1^ at the current density of 1 A g^-1^, 73 W h Kg^-1^, 1056 W kg^-1^, and 89% after 2000 cycles, respectively. The incorporation of MC increases the porous structure and contact of electrolyte ions with the electrode. These enhance the electrochemical properties of the supercapacitor [70]. Cellulose acetate (CA), chitosan (CS), rGO, NiO, and Fe_3_O_4_-based electrode was prepared by phase inversion and dip-polymerization technique [71]. The PVA/NaNO_2_ gel electrolyte was used for analysing electrochemical properties. The areal capacitance and capacitance retention were 16.61 mF cm^-2^ at a scan rate of 5 mV s^-1^ and 88% after 1000 cycles. The morphological structure of the electrode provides the unique electrochemical performance of the supercapacitor [71].

##### 5.1.1.2 Nanostructural cellulose

**Fig. 6:**
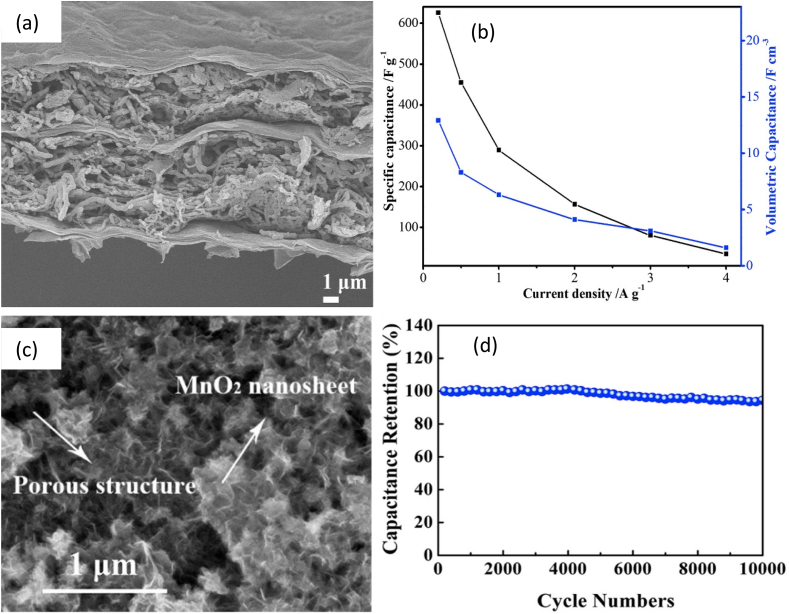
(a) SEM image of CNF/rGO/PPy nanocomposite electrode [73]; (b) Specific capacitance of CNF/rGO/PPy based supercapacitor [73]; (c) SEM image of NC/MnO2 nanocomposite electrode [89]; and (d) Capacity retention of NC/MnO_2_ based supercapacitor [89].

CNF and CNT based nanohybrid (CNT/CNF) was mixed with polyvinyl alcohol-borax (PVAB) hydrogel to develop CNF/CNT/PVAB bionanocomposite as an electrode (cathode and anode) for supercapacitor. CNF/PVAB hydrogel was used as an electrolyte. CNF/CNT/PVAB showed a specific capacitance of 117.1 F g^-1^ and capacitance retention of 96.4% after 1000 cycles [72]. Vacuum filtration was also applied to prepare CNF/graphite and MFC/graphite-based bionanocomposites as electrodes. MFC/graphite-based supercapacitor has shown superior electrical properties compared to CNF/graphite-based supercapacitor. The dispersion of graphite with MFC leads to provide better electrical properties [18]. In another study, CNF, rGO, and PPy were used to produce CNF/rGO/PPy-based nanocomposite (Fig. 6a) using vacuum filtration and chemical reduction methods. 1.0 M H_2_SO_4_ was used as an electrolyte. CNF/rGO/PPy based supercapacitor had shown specific capacitance of 625.6 F g^-1^ at 0.22 A g^-1^ Fig. 6b, and capacitance retention of 75.4% after 5000 cycles, respectively. It had an energy density of 21.7 Wh kg^-1^ at a power density of 0.11 kW kg^-1^ [73]. Again, lignin-containing cellulose nanofibrils (LCNF)-based bionanocomposite was prepared by making a film of LCNF/rGO followed by coating with PANI using in situ polymerization technique to obtain LCNF/rGO/PANI bionanocomposite as an electrode [74]. The filtration method was used to obtain NC/rGO/PANI bionanocomposite as an electrode for a supercapacitor and it exhibited a power density of 147.53 W kg^-1^ and an energy density of 5.09 Wh kg^-1^. The presence of rGO and PANI enhances capacitance and conductive properties [30].

Aerogel-based bionanocomposites have been developed as an electrode for a supercapacitor. CNC/PPy aerogel was made by freeze-drying as an electrode for the supercapacitor. The supercapacitor showed a charge retention capacity of 61.66–84.19% after 2000 cycles. The presence of a large surface for capacitive material helps store the charge [75]. In another study, a mixture of CNF and vapour-grown carbon fiber (VGCF) was freeze-dried followed by the polymerization of PPy to obtain CNF/VGCF/PPy aerogel. CNF/VGCF/PPy aerogel had a capacitance of 678.66 F g^-1^ and capacitance retention of 91.38% after 2000 cycles. The VGCF and PPy have double-layer capacitance and pseudocapacitance, respectively. These contribute to excellent electrochemical properties of CNF/VGCF/PPy aerogel-based supercapacitor electrodes [76]. Again, PANI penetrated CNF aerogel followed by carboxylic multiwalled carbon nanotubes (CMWCNTs) using vacuum filtration to obtain CNF/PANI/CMWCNTs aerogel electrode. A similar process was applied to produce CNF/PANI/GO aerogel electrodes. Those electrodes were autoclaved to reduce to obtain better electrochemical properties. CNF/PANI/CMWCNTs and CNF/PANI/GO aerogel electrodes showed capacitance of 965.80 and 780.64 F g^-1^, respectively. The energy density was 147.23 and 112.32 mWh cm^-2^ for CNF/PANI/CMWCNTs and CNF/PANI/GO aerogel-based supercapacitors, respectively. An interconnected multilayer structure with hydrophilic nature provides a high surface area and contact area along with ion accessibility. These attributes have excellent electrochemical properties [77]. Researchers also made CNF/PANI aerogel as an electrode for a supercapacitor with a capacitance of 291.01 F g^-1^. PANI provides structural support to the aerogel and facilitates electron transfer leading to excellent capacitance [78].

LBL technique has been applied to produce the electrode for a supercapacitor. Polyelectrolytes (PE) functionalized CNF aerogels beads and carboxyl-functionalized single-wall carbon nanotubes (CF-SWCNTs) were used to obtain CNF/PE/CF-SWCNTs bionanocomposite by LBL technique for a supercapacitor [79]. Similarly, CNF/CNT bionanocomposites were produced [80]. The authors made a top and bottom layer with a mixture of CNF and CNT suspension by filtration using a cellulose ester membrane, and the middle layer was CNF suspension. CNF/PE/CF-SWCNTs composite materials have shown electrical conductivity of 2 kA cm^-2^ and a charge storage capacity of 9.8 F g^-1^ [79]. Again, CNT, anhydrous cobalt (II) chloride (CoCl_2_), and polyvinylpyrrolidone (PVP) were used to make PCC composite products by calcination at N_2_ before developing NC/PCC bionanocomposite by vacuum filtration. In this composite, the PCC layer was at the bottom and the NC layer was at the top. NC/PCC-based electrodes had a capacitance of 93.75 mF cm^−2^. The uniform distribution of CNT in the matrix improves conductivity [81]. Furthermore, the presence of Co enhances the electrochemical properties [82].

Modification of NC has been done to improve the properties of electrodes for the supercapacitor. CNF was modified as cationic and anionic CNF. The modified CNF and PPy-based bionanocomposites (CNF/PPy) were produced by forming PPy on CNF through chemical polymerization [83]. A blend of sulfonated CNT and TEMPO-oxidized CNF was moulded to produce CNF/CNT hydrogel followed by the polymerization with PPy to obtain CNF/CNT/PPy nanocomposite as an electrode [84]. TEMPO oxide NC, CNT, pigskin (PS) powder, and glutaraldehyde-based NC/CNT/PS electrode for the supercapacitor were prepared by sonication, heating, and agitation. It has a specific capacitance of 65 F g^-1^ and capacitance retention at around 60% after 2000 cycles in 1.0 M H_2_SO_4_ as an electrolyte. NC helps to distribute CNT uniformly in the PS matrix leading to getting continuous conductive pathways [85].

A carbonization procedure has been used to produce cellulose-based bionanocomposite as an electrode for the supercapacitor. NC/GO aerogels have been carbonized to obtain the electrode having a specific capacitance of 224 F g^-1^ at the current density of 1 A g^-1^ and capacitance retention of 97% after 100 cycles [86]. Similarly, bio-Ac, rGO, and CNF-based aerogel have been used as self-supporting CNF/bio-Ac/rGO-based electrodes, and this electrode-based supercapacitor had a high capacitance of 812.2 mF cm^-2^, a long cycling life, and an energy density of 0.365 mW h cm^-2^ [87]. The presence of pores, holes at the edge, and CO-type functional groups enhance to obtain the electrochemical performance [86]. In another study, skin secretion of *Andrias davidianus* (SSAD), CNC, and CNF have been used to produce honeycomb structural SSAD/CNC/CNF electrodes by freeze-drying followed by carbonization. Supercapacitors composed of this electrode had a high capacitance, cycling stability, and charge density. SSAD works as the source of N_2_ to improve the electrochemical properties [88]. Again, a mixture of NC and the cleaned dandelion fluffs were carbonized to obtain porous carbon nanosheets (PCNs). The PCNs were mixed with MnO_2_ followed by microwave irradiation, washing, and drying to produce a PCNs/MnO_2_ bionanocomposite electrode (cathode) (Fig. 6c) for a supercapacitor. PCNs/MnO_2_-based supercapacitor had shown an energy density of 28.2 Wh kg^-1^, a power density of 899.36 W kg^-1^, and capacitance retention of 89% after 10000 cycles (Fig. 6d) in 1.0 M Na_2_SO_4_ as an electrolyte. The porous structure and GE-like structure enhance electrochemical properties through electron transfer and electrolyte permeation [89]. In another investigation, CNF and activated carbon (AC) mixture was used to make a CNF/AC film followed by carbonization to obtain CNF/AC-based supercapacitor [90]. Furthermore, a pyrolysis technique was applied to a freeze-dried mixture of MoS_2_, CNF suspension, and GO suspension for preparing a CNF/MoS_2_/rGO bionanocomposite as an electrode for a supercapacitor [91]. As a coating process, carbonized CNF aerogel was coated on the nickel (Ni) substrate to obtain CNF/Ni electrode for a supercapacitor and it showed an energy density of 4.5 Wh kg^-1^ when 6.0 M KOH was used as electrolyte [92].

#### 5.1.2 Bacteria oriented cellulose-based bionanocomposites as electrode for supercapacitor

##### 5.1.2.1 Microstructural cellulose

**Fig. 7:**
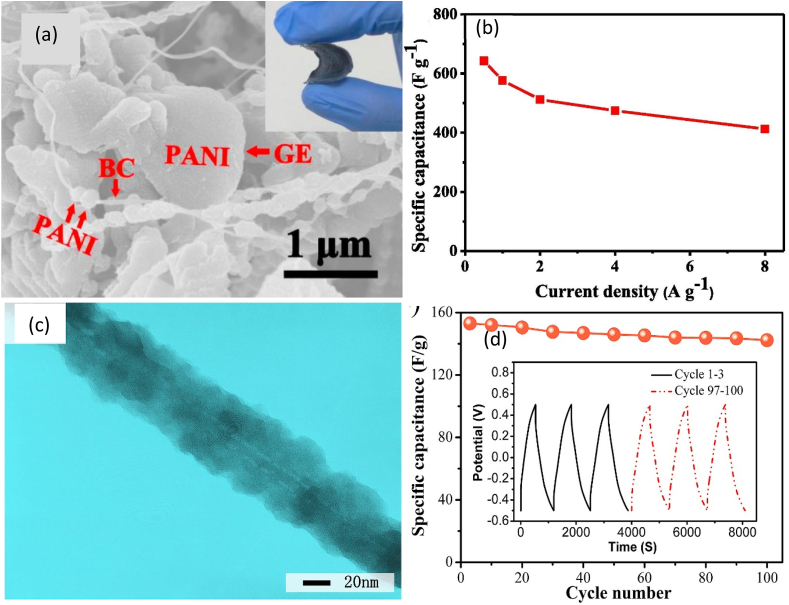
(a) SEM image of BC/GE/PANI bionanocomposite [95]; (b) Variation of specific capacitance with current density of BC/GE/PANI bionanocomposite-based supercapacitor [95]; (c) TEM image of BNC/PPy bionanocomposite [100]; and (d) cycling stability of BNC/PPy bionanocomposite-based supercapacitor [100].

BC, AgNPs, and PANI-based bionancomposites (BC/AgNPs/PANI) were prepared through in situ polymerization followed by a freeze-drying technique [93]. The use of BC/AgNPs/PANI as cathode and anode electrodes in a supercapacitor using 1.0 M H_2_SO_4_ as electrolyte showed an energy density of 34 Wh kg^−1^ at a power density of 459 Wh kg^−1^. The presence of a mesoporous structure with a high specific surface ensured excellent electrochemical properties [93]. Wan et al. [94] proposed the LBL technique to develop BC, GE, and PANI-based bionanocomposite (BC/GE/PANI) for application in an electrode. In another study, Luo et al. [95] applied the LBL technique to obtain BC/GE/PANI bionanocomposites as electrodes of supercapacitor (Fig. 7a). The authors used in situ culture of BC by LBL followed by dispersion of GE nanosheet in BC, polymerization of PANI, and freeze-drying. Cathode and anodes were used as BC/GE/PANI and the electrolyte was 1.0 M H_2_SO_4_. The obtained energy density was 14.2 Wh kg^−1^ at a power density of 200 W kg^−1^ and gravimetric capacitance was 645 F g^−1^ at 1 A g^−1^ (Fig. 7b). The good distribution of GE nanosheets in the highly porous 3D BC network and deposition of PANI on the surfaces of both BC and GE nanosheets enhance the electrochemical properties of the supercapacitor [95].

##### 5.1.2.2 Nanostructural cellulose

For the fabrication of the BNC-based electrode, a thin film composed of BNC, SnO_2_ nanoparticles, and rGO was coated with PEDOT and poly(styrenesulfonate) solution (PEDOT:PSS) to obtain BNC/SnO_2_/rGO/PEDOT:PSS bionanocomposite and it showed capacitance of 445 F g^-1^ and capacitance retention of 84.1% after 2500 cycles [96]. In another study, nanosized MnO_2_ was impregnated into BNC membranes followed by the polymerization of PPy with the addition of CuCl_2_.2H_2_O, washing, and drying to obtain BNC/MnO_2_/PPy/CuCl_2_ nanocomposite as an electrode for supercapacitor [97]. Wesling et al. [98] produced BNC/PPy/CuCl_2_ nanocomposite as an electrode for supercapacitors following a similar method without MnO_2_. In another study, Ma et al. [99] produced BNC/PPy/rGO bionanocomposites by in situ polymerization and vacuum filtration. These were also used as cathode and anode electrodes in a supercapacitor using 1.0 M NaNO_3_ as electrolyte and it showed a capacitance of 1.67 F cm^-2^ and an energy density of 0.23 mW h cm^-2^ at a maximum power density of 23.5 mW cm^-2^. Porous structure and accessible surface area allow for penetrating electrolytes leading to excellent electrochemical properties [99]. In another study, TEMPO-oxidized BNC was used to produce BNC/PPy bionanocomposites (Fig. 7c) and these were used as cathode and anode electrodes in a supercapacitor cell [100]. The authors used 1-Ethyl-3-methylimidazolium tetrafluoroborate (PVDF-EMIMBF4) polymer as an electrolyte and the observed specific capacitance, energy density, and capacitance retention were 153 F g^-1^, 21.22 Wh kg^-1^ at the current density of 0.2 A g^-1^, and about 93% after 100 cycles, respectively (Fig. 7d). These electrodes had shown good bending stability because of NC in the matrix [100].

### 5.2 Electrolyte of plant oriented cellulose-based bionanocomposites for supercapacitor

CMC, chitosan (CS), and CNT were used to prepare the nanocomposite electrolyte of CMC/CS/CNT using a sol-gel and drying process [31]. The observed specific capacitance was 94 F g^-1^ at a current density of 100 μA cm^-2^. The low current leakage and diffusion criteria of this electrolyte provide excellent electrochemical properties of the supercapacitor [31].

## 6 Challenges and opportunities of cellulose-based bionanocomposites in energy storage applications

The use of cellulose-based bionanocomposites in energy storage applications is a new dimension for the sustainable supply of energy. Researchers have also faced trouble to obtain a satisfactory level of performance for certain types. BC/Ag/PANI aerogel as a flexible and lightweight electrode has not shown enough electrochemical performance for energy storage applications and improvement is needed using suitable modification techniques [93]. For this, carbonization of BC can be applied to develop the electrode since carbonized BNC can increase the conductivity and lithium storage [63]. Researchers have observed improved properties of the electrode when gum [101] and polyimide (PI) [102] are used as binders instead of CMC. Chen et al. [101] reported that CMC/Si/GE bionanocomposite as an electrode for LIB showed lower electrochemical properties compared to gum/Si/GE-based nanocomposite. However, using CMC as a binder can help to fabricate the electrode economically and environmentally friendly [48,103]. Again, the Si-based anode has a high capacity [104] but it has a problem with high volume change during the charge-discharge process [29]. The use of CF-based bionanocomposites for developing electrodes can solve the problem [29]. Electrode materials need to be improved for obtaining the high performance of LIB [105]. In addition, cellulose-based bionanocomposites can provide low-cost and lightweight [106], biodegradable, and mechanically flexible [107] energy storage devices. On the other hand, NC-based bionanocomposite can provide self-healing ability, malleable, and strong energy storage devices [72].

Moreover, a high aspect ratio and specific surface area of BC can promote its use as functional material in energy storage applications, and it can grow economically in the industry through a microbial fermentation process [108]. Again, electrodes can be made from BC-based bionanocomposites more easily. Thus, the economical and recyclable energy storage devices can be developed on a large scale [100].

## 7 Conclusions

Cellulose-based bionanocomposites are promising to employ for the development of energy storage devices. In general, these are made in combination with either organic or inorganic materials. Researchers have put their immense intention to develop environmentally-friendly batteries and supercapacitors from these types of advanced hybrid materials. Bionanocomposites derived from plant-based cellulose are used for developing electrodes, separators, and collectors for batteries while electrodes and separators are prepared for supercapacitors. On the other hand, bacterial cellulose is used for making an electrode for batteries and supercapacitors. However, bacterial cellulose is promising because of its availability, easier production, and smooth application in an energy storage device. Cellulose is used as either a binder or reinforcing material for manufacturing the component of energy storage devices. Carboxymethyl cellulose (CMC) is widely used as a binder but it has been claimed that CMC has less performance in terms of electrochemical properties compared to other binders. Nevertheless, CMC is extracted from available renewable biomaterial and its performance can be improved by incorporating other materials. Furthermore, cellulose-based bionanocomposites can provide flexible, lightweight, biodegradable, and strong energy storage devices for the present and next generation. Continuous research can help develop bio-based energy storage devices to overcome environmental issues and obtain sustainability in the energy sector.

## Author contribution statement

All authors listed have significantly contributed to the development and the writing of this article.

## Funding statement

This research did not receive any specific grant from funding agencies in the public, commercial, or not-for-profit sectors.

## Data availability statement

No data was used for the research described in the article.

## Declaration of competing interest

The authors declare no conflict of interest.
